# Passage of a Large Stone through the Urethra

**Published:** 1860-11

**Authors:** W. M. Turner

**Affiliations:** Petersburg, Va.


*Passage of a Large Stone Through the Urethra of a Boy two Years old.* Reported by W. M. Turner, M. D., of Petersburg, Va.

On the night of the 29th ult., I was sent for to see a boy who, the woman told me, was suffering from retention of urine.—The case was under the treatment of Dr. Weldon Claiborne, of this city, and the woman who called me brought a request from Dr. C. to carry a catheter with me and endeavor to get away the urine; he, the Doctor, being compelled to go into the country before returning to his office, and he had, unfortunately, no catheter with him. I went, and found for my patient a handsome little boy (colored), about 27 months old. He was lying on his back, moaning most piteously—his countenance betraying acute suffering. The pulse was both frequent and quick, ranging in beats to the minute from 125 to 135. Before proceeding to explore the region of the bladder, I was led to examine the chest by the circumstance of accidentally placing my hand over the right lung, and feeling a decided vibratory thrill. Applying my ear for auscultating purposes, I detected a pneumonia in the upper lobes of both lungs, and in the *second* stage; as bronchial and bronchophony were well marked, as well as was dullness on percussion. Upon inquiry, I learned that the child had been sick a month previous, with measles, and, having taken cold when recovering, had been complaining more or less ever since that time.

The bladder, to which I then turned my attention, was enormously distended, protruding and hanging over the public arch, semewhat resembling, in miniature, the "*pendulous-belly*," of pregnant women. The epidermis of the abdomen, over the bladder, was quite red and shining, and I was indeed fearful of an artificial opening. The penis was much enlarged, and apparently infiltrated with urine. This, however, was not the case, as subsequent examination proved. To add to the difficulties of the case, there was present a congenital phymosis—rendered ten times more rigid in its contraction by the swollen state of the penis. It was a difficult task, which I then set to work to accomplish—that of drawing off the urine of a boy 27 months old, under circumstances of such pain to the little patient, and of such disadvantage to myself. The swollen and phymosed condition of the penis, and the great irritability of the child, rendered it almost impossible even to make an attempt with the catheter. At the slightest touch the boy would shriek and scream, until I actually thought the strained bladder would rupture before the prolonged muscular contraction would relax. Finally, after much patience and considerable manœuvring, my only assistant being an old lady, who managed to hold the child's hands, I succeeded in passing a small gum catheter through the hard, pursed-up, phymosed ring, and, as fortune would have it, into the urethra. This *feat* I was compelled to perform several times, for the frantic efforts of the child dislodged the catheter, and my labor was thrown away. However, I placed my knee upon the lower extremities of the child, and in this position succeeded in passing the instrument once more into the urethra. Moving it slowly and cautiously downward, I soon had the satisfaction of reaching the bladder, which I knew to be the case by the sudden gush of the pent-up urine through the catheter. At the neck of the bladder, I noticed a slight *gritty* feel as the catheter glided along; it was transient, however, and I thought nothing of it, attributing it to the ring of a cartilaginous stricture, which I had no doubt existed. The boy was instantly relieved, and fell asleep while the urine—an enormous quantity—was flowing. I prescribed nothing for the patient that night, except cold cloths around the penis. The next morning, the first of August, I saw the boy with Dr. Claiborne; we found the patient much refreshed and considerably improved by a good night's rest. The belly, over the region of the bladder, had lost the prominent *bullety* appearance of the previous evening, and appeared more natural, yet it was still very tender. The penis possessed yet its infiltrated look, and resembled much a transparent gelatinous substance—differing only in a *solid appearance.* We found that the boy had already passed his urine once, but in a very unsatisfactory manner, screaming at the top of his voice when the urine reached the urethral canal, and refusing most positively to make further effort.— The result was, when we arrived, he was again unable to urinate. Once more my little gum catheter was called into requisition, and our united efforts had the desired effect of bringing away the urine. As I had a case of instruments with me, Dr. C. advised operation for phymosis; suggesting that, perhaps, the rigidity with which it fastened aronnd the glans penis prevented micturition. We divided the pressure with a bistoury and director. The operation was over in a few minutes. After splitting the prepuce, we discovered that the retention of the urine could not have been due to the pressure made over the mouth of the urethra by the phymosis. When I passed the catheter in to empty the bladder, I detected, very plainly, the same *gritty* feel which I had remarked the evening previous. Dr. Claiborne was equally sensible of the peculiar sensation. The touch was decisive; there could be no mistaking the fact of the presence of a foreign body. Dr. Claiborne immediately pronounced the case to be stone. In withdrawing the catheter, which I had used as a sound, I distinctly felt an opposing substance. Dr. C. at this moment remarked, that he distinctly felt, between his thumb and forefinger, a round or irregularly round tubercle in the urethra. After several introductions of the catheter, I finally succeeded in hooking the stone in the fenestra of the instrument, and, with a sudden motion, drew it forward an inch toward the external orifice of the *meatus urinarius.* Here we experienced much difficulty in our work; we dared not split the urethra; and yet it was very difficult to obtain a hold on the stone, which showed a constant tendency to retrocede. Finally, after much patience and manipulation, we obtained a fastening on the stone with a pair of dressing forceps, and extracted it.— The boy came near swooning, but soon revived. The following are the dimensions of the stone: Length five eights, and circumference three-quarters of an inch; weight, nearly forty-two grains; shape, semi-conoidal and semi-cylindrical —the presenting end corresponding to the apex of the cone. An analysis of the stone proved it to be oxalate of lime.

A further examination, using the catheter as a sound, revealed, as we had already suspected, the presence of another calculus in the bladder, and we came to the conclusion that the lithogenesis was hereditary. On inquiry, howrver, I learned nothing which would warrant the supposition. The boy has not suffered, especially since the time of the passage of the stone. He is now running about, and seems to be lively enough. That he will be troubled again with calculus, there can be no reasonable doubt; how soon first, I cannot say.

The noticeable feature in this case is, that a *stone of such a size* did pass through the urethra of a child two years of age.—*Maryland and Virginia Medical Journal.*

